# Molecular surveillance of malaria in Tanzania: Baseline surveillance of vectors that drive persistent malaria transmission in regions with varying transmission intensities

**DOI:** 10.1371/journal.pone.0346692

**Published:** 2026-04-15

**Authors:** Yahya A. Derua, Bernard M. Batengana, Filbert Francis, Celine I. Mandara, Rashid A. Madebe, Misago D. Seth, Daniel P. Challe, Deus S. Ishengoma

**Affiliations:** 1 National Institute for Medical Research, Amani Research Centre, Muheza, Tanga, Tanzania; 2 National Institute for Medical Research, Tanga Research Centre, Tanga, Tanzania; 3 National Institute for Medical Research, Dar es Salaam, Tanzania; 4 Ifakara Health Institute, Dar es Salaam, Tanzania; Fundação Oswaldo Cruz Centro de Pesquisas René Rachou: Fundacao Oswaldo Cruz Instituto Rene Rachou, BRAZIL

## Abstract

Malaria has remained persistently high in some regions of Tanzania despite increased control interventions. To address the burden of malaria in these regions, monitoring malaria vector dynamics is crucial to inform control interventions. This study assessed the composition and infectivity of malaria vectors in areas with varying levels of transmission. A cross-sectional study was conducted in five districts: Buhigwe, Kyerwa, Ludewa, Muheza, and Nyasa, from August to October 2023. In each district, one to five villages were selected for the study, and mosquitoes were collected indoors from 10 households per village using CDC light traps. Outdoor mosquitoes were collected from five households using Furvela tent traps. In all selected villages and trap types, mosquitoes were collected over three nights. Collected mosquitoes were sorted by species, and malaria vectors were sent to the laboratory for identification and screening for malaria parasites using polymerase chain reaction. A total of 19,898 mosquitoes were collected, and *Anopheles gambiae* complex, *An. funestus* group, other *Anopheles* species and culicine species accounted for 2.2%, 44.3%, 1.1% and 52.4%, respectively. *An. funestus* group was the predominant species, accounting for 95.3% of the malaria vectors. Sibling species identification revealed that *An. arabiensis* was the main species (75.0%) in *An. gambiae* complex, whereas *An. funestus* sensu stricto (s.s) was predominant (96.3%) in *An. funestus* group. A total of 1799 vectors were tested for infection with *Plasmodium falciparum,* and nine (0.5%) collected with CDC light traps were infected. The estimated annual entomological inoculation rate for *An. funestus* collected by light traps was 242.6, 66.5, and 13.0 infectious bites per person per year in Kyerwa, Nyasa, and Muheza, respectively. This study recorded a relatively high density of malaria vectors, particularly in Kyerwa district, despite being conducted during the dry season. *An. funestus* was the main vector, and interventions targeting it are urgently needed to achieve the national goal of malaria elimination by 2030.

## Background

Malaria remains an important mosquito-borne parasitic disease of high public importance as a cause of high morbidity and mortality, especially among non-immune groups such as infants and pregnant women. According to the 2024 World Malaria Report, there were an estimated 263 million cases of malaria and 597,000 deaths globally in 2023 [1]. Of the reported global deaths, four countries accounted for just over half of all malaria deaths worldwide: Nigeria (31%), the Democratic Republic of the Congo (11%), Niger (6%), and the United Republic of Tanzania (4%) [1].

In sub-Saharan Africa (SSA), malaria is mainly caused by *Plasmodium falciparum* (over 90% of cases) and is transmitted by members of the *Anopheles gambiae* complex and *An. funestus* group [2]. Recently, an invasive Asian malaria mosquito, *An. stephensi* has also been reported in some countries in Africa, including Sudan, Somalia, Nigeria, Eritrea, Kenya, and Ghana [3] and is involved in malaria transmission in Djibouti and Ethiopia [4,5]. In Tanzania, *An. gambiae* sensu stricto (s.s), *An. arabiensis*, and *An. funestus* s.s are the main malaria vectors with a wider geographical distribution [6]. *An. gambiae* s.s and *An. arabiensis* vary in their geographical distribution, but the sympatric occurrence of the two-sibling species is common [7]. A new cryptic taxonomic group named the Pwani molecular form within *An. gambiae* complex has been identified along the coast of Tanzania [8]. Several studies have reported the re-emergence of *An. funestus* as an important malaria vector in Tanzania and other SSA countries [9–11].

Malaria vector control in SSA relies on the use of long-lasting insecticide-treated nets (LLINs) and indoor residual spraying (IRS) [12]. Currently, LLINs are the only core malaria vector control intervention deployed throughout mainland Tanzania, including the current study sites. However, these interventions are becoming increasingly threatened by insecticide resistance, affecting classes of insecticides used for public health [13]. In the past, pyrethroid-only LLINs were recommended by the World Health Organization (WHO) for deployment as a core intervention in all malaria-endemic settings. In 2019, the WHO prequalified pyrethroid plus a synergist piperonyl butoxide (PBO), for deployment in malaria-endemic settings where the principal vectors exhibit pyrethroid resistance [12]. Recently, the WHO has provided a strong recommendation for the deployment of pyrethroid-chlorfenapyr instead of pyrethroid-only LLINs in areas with pyrethroid resistance [14]. In Tanzania, the prevalence and intensity of malaria vector resistance to pyrethroids have expanded over the years, covering large geographical areas [15]. Moreover, insecticide resistance has also been recorded in bendiocarb (carbamate), a class of insecticides used in IRS [15]. Furthermore, it has also been shown that the susceptibility of different mosquito vectors to LLINs varies owing to their diverse feeding and resting behaviors. A study conducted in northeastern Tanzania showed that LLINs were more effective at controlling *An. gambiae* s.s than *An. arabiensis* in experimental hut settings [16]. It has been shown that widespread insecticide resistance threatens the success of malaria control achieved through large-scale implementation of LLINs and IRS over the last decade [14].

LLINs and IRS become protective only if mosquito vectors enter human dwellings to seek a blood meal, and possibly rest indoors. However, it has been well documented that mosquito vectors in different SSA settings have changed their feeding and resting behavior, making it difficult to control using methods that target indoor feeding and resting mosquitoes [17,18]. Moreover, studies have shown that the composition of *An. gambiae* complex has changed in different ecological settings where LLINs and IRS have been deployed [19–21]. In Tanzania, *An. arabiensis* has become the predominant malaria vector in different geographical settings, where *An. gambiae* s.s and *An. arabiensis* coexisted [10,19]. *An. arabiensis* is known for its flexible behavior, including biting and resting indoors or outdoors, and switching its host from humans to domestic animals [7]. Furthermore, it has been shown that outdoor biting and resting malaria vectors are capable of maintaining malaria transmission in areas with high coverage of LLINs and IRS intervention [22–24].

Despite the intensification of malaria control interventions, such as vector control, case management focusing on prompt diagnosis and treatment with effective antimalarial drugs, preventive therapy, and community education strategies to promote disease control measures, malaria has remained persistently high in some areas of eastern, northwestern, and southern regions of Mainland Tanzania. The 2022 Tanzania malaria indicator survey showed a slight increase in malaria prevalence in children aged 6–59 months from 7.2% in 2017 to 8.0% in 2022 [25]. This calls for more research on the epidemiological, entomological, and human-related factors that drive persistent malaria transmission in mainland Tanzania. Malaria vector entomological surveillance conducted in 32 district councils in mainland Tanzania has reported an increasing role of *An. arabiensis* and *An. funestus* s.s in malaria transmission [26]. To guide the deployment of appropriate vector control interventions, it is crucial to strengthen and expand entomological surveillance activities to target new sites and incorporate novel surveillance tools.

Thus, the current study was designed and implemented as part of a project on molecular surveillance of malaria in Mainland Tanzania (MSMT), which was initially focused on malaria parasites (in 2021 and 2022) [27,28] but was later expanded to incorporate genomic surveillance of malaria vectors as part of the efforts for integrated malaria molecular surveillance (iMMS) [29]. This study aimed to provide baseline data on the composition and infectivity of malaria vectors that maintain malaria transmission in selected MSMT regions with varying endemicity, thereby building the capacity for vector genomics within the ongoing iMMS in Tanzania. The current study expanded the countrywide malaria vector entomological surveillance sites to newer sites for a better representation of mainland Tanzania. Establishing and strengthening the iMMS, which covers parasites, vectors, and human hosts, will provide important information to guide the design and implementation of appropriate malaria control interventions with better targeting of the vectors to achieve the country’s target of malaria elimination by 2030.

## Materials and methods

### Study area and design

**Fig 1:**
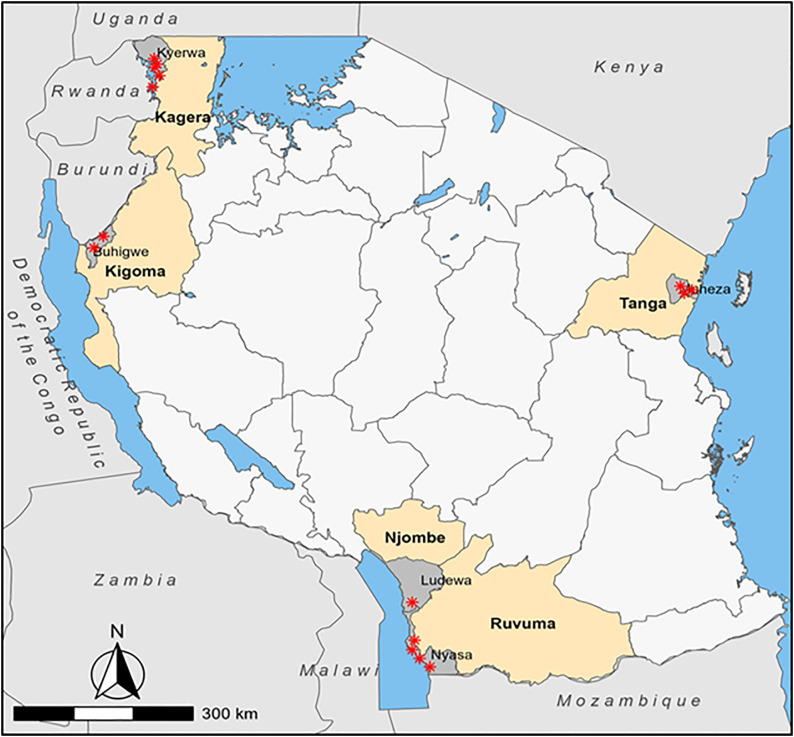
Map of Tanzania showing study regions (in ivory), districts (in grey), and villages (in red stars): Republished with permission from Springer Nature [30].

This study was conducted in five districts of mainland Tanzania with varying levels of malaria transmission: Buhigwe (Kigoma Region), Kyerwa (Kagera Region), Ludewa (Njombe Region), Muheza (Tanga Region), and Nyasa (Ruvuma Region) (Fig 1). The study was conducted from 30^th^ August to 26^th^ October 2023, and the sites covered were involved in a countrywide iMMS conducted by the MSMT project in 2023 [30]. In each district, a variable number of villages (ranging from one to five) were selected for malaria vector surveillance based on their involvement in the community surveillance components of the MSMT project, as previously described [30,31].

In brief, the surveyed villages were characterized by rural ecosystems with limited infrastructure, low population density, and inhabitants mainly relying on subsistence farming and animal husbandry. Buhigwe District is located in northwestern Tanzania. The district experiences warm temperatures, averaging 25°C, and rainfall ranges from 750 to 1000 mm, mostly occurring between November and May [32]. Kyerwa District lies in the Lake Victoria basin and borders Uganda to the north and Rwanda to the west. The district experiences warm temperatures, averaging 24–32°C, and receives between 1750 and 2000 mm of rain annually. Rainfall peaks twice a year, with heavy rains from March to May and shorter ones from October to December [32]. Ludewa District is situated in the Southern Highlands and experiences cooler temperatures, averaging 21–24°C. Its annual rainfall ranges from 750 to 1000 mm, mostly falling between November and April. Muheza District, near the Indian Ocean coast, has a hot and humid climate with average temperatures ranging from 24 to 34°C. The annual rainfall varies from 1000 to 1250 mm, with two rainy peaks from March to May and a shorter period from October to December [32]. Additionally, Nyasa District, located in southern Tanzania along Lake Nyasa, borders Mozambique to the south and Malawi to the west. Rice farming and fishing are key economic activities in this district. It experiences warm temperatures, averaging 26°C, and annual rainfall ranges from 1000 to 1250 mm, mostly falling between November and April [32].

**Fig 2:**
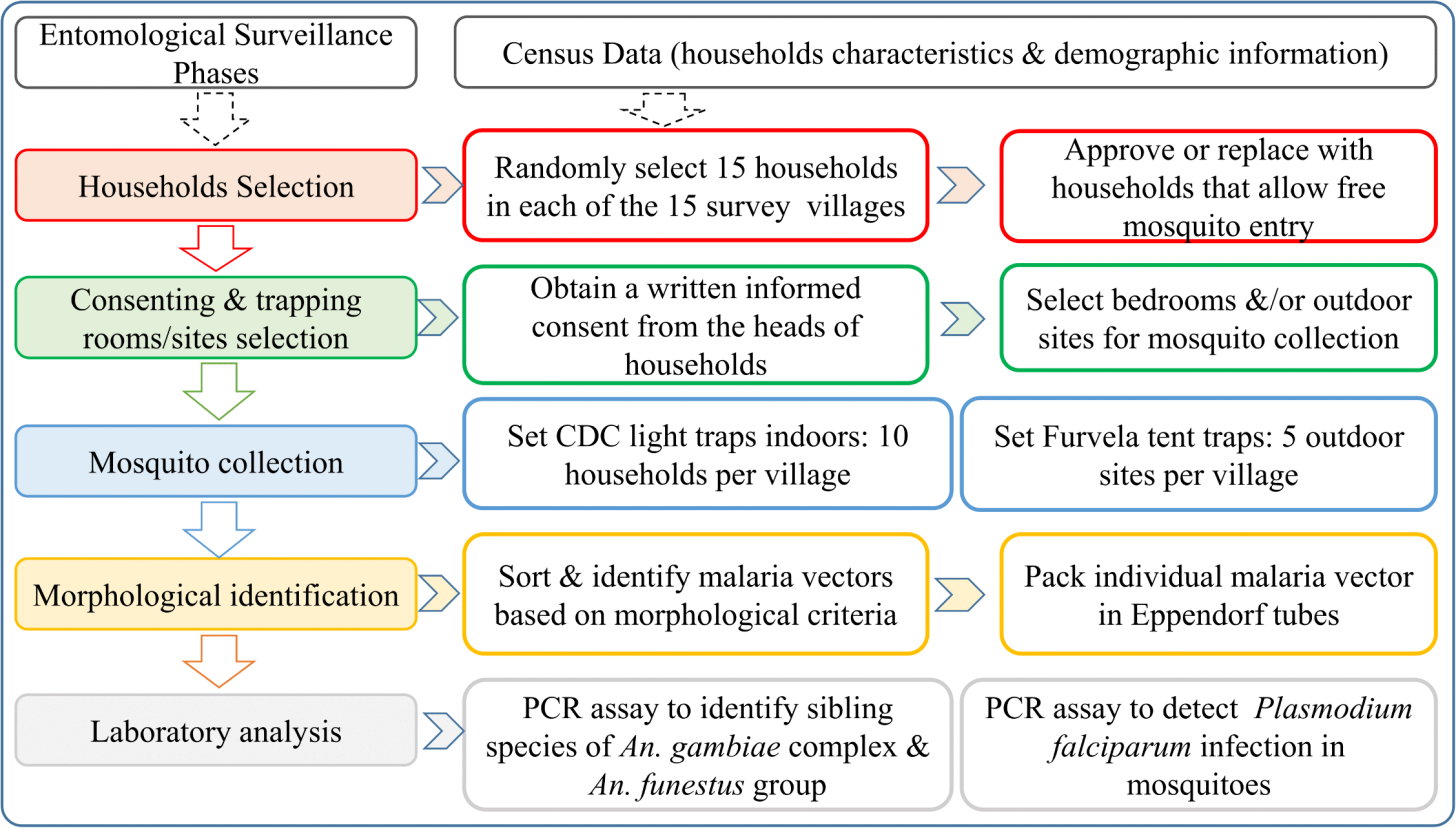
Study design and activities undertaken during the entomological survey.

Ten and five households were randomly selected from each of the selected villages for sampling indoor and outdoor host-seeking malaria vectors, respectively (Fig 2). A list of all households in the study villages was created using the census survey, which was conducted by the MSMT project as previously described [30]. The list was used to randomly select trapping households, ensuring wider coverage and representation of all parts and hamlets in the village. Efforts were made to select trapping households with open eaves or other open spaces to allow free mosquito entry and exit from the houses. All selected trapping houses were mapped using a Global Positioning System (GPS) application running on Android smartphones.

### Mosquito collection

#### Indoor mosquito collection.

In each village, indoor mosquitoes were collected for three consecutive nights in 10 selected houses using Centers for Disease Control (CDC) light traps (John W Hock Co, Gainesville, FL, USA). Before mosquito collection, written informed consent was obtained from the head of the trapping households. CDC light traps were set at the foot end of the bed occupied by a family member sleeping under LLIN as previously described [33]. In brief, the traps were set between 18.00 and 19.00 hours and retrieved the following morning between 06:00 and 07:00 hours.

#### Outdoor mosquito collection.

From each study village, outdoor mosquito collections were conducted for three consecutive nights in five selected houses using Furvela tent traps. The Furvela tent trap consisted of a tent and a CDC light trap (light bulb removed) positioned in close proximity to selected houses. To attract host-seeking mosquitoes, a volunteer slept in a tent with a small opening at the tent door, allowing the host odor to escape, as previously described [34]. The tent traps were set each night between 18:00 and 19:00 hours and retrieved the following morning between 06:00 and 07:00 hours.

### Morphological identification of mosquitoes

The collected mosquitoes were transported to the field laboratory, which was set up in each village, sorted, and malaria vectors were identified to the species level based on morphological criteria [35]. Malaria vectors were further classified according to their abdominal status as unfed, blood-fed, semi-gravid, or gravid. *An. gambiae* complex, and *An. funestus* group were stored individually in Eppendorf tubes containing silica gel desiccants for further molecular analysis. All other *Anopheles* species and culicine mosquitoes were identified to the genus level, counted, and discarded.

#### Molecular identification of sibling species of *An. gambiae* complex and *An. funestus* group.

In the laboratory, DNA was extracted from the head and thorax of a random sample of malaria vectors (*An. gambiae* complex and *An. funestus* group) using the Chelex-100 method [36]. Members of *An. gambiae* complex were identified to their respective sibling species by polymerase chain reaction (PCR) based on species-specific single-nucleotide polymorphisms (SNPs) in the intergenic spacer region (IGS) on the ribosomal DNA [37]. In brief, PCR reactions were conducted in a final volume of 20 μL consisting of 0.3 μM of each of the five primers (universal, *An. gambiae* sensu stricto (s.s), *An. arabiensis*, *An. merus*, and *An. quadriannulatus*), 1:1 TEMPase HotStart Master Mix (Ampliqon-Denmark), and 3 μL DNA template. The samples were amplified on a Bio-Rad T100 thermocycler (Bio-Rad, Singapore) with cycling conditions of pre-heating at 95°C for 15 min, followed by 30 cycles of denaturation at 94°C for 30 s, annealing at 50°C for 30 s, extension at 72°C for 30 s, and a final extension at 72°C for 5 min.

The sibling species of *An. funestus* group were identified by PCR using a previously described method targeting species-specific SNPs in the internal transcribed spacer region 2 (ITS2) on the ribosomal DNA [38]. Each PCR run was conducted in a final volume of 20 μL consisting of 0.3 μM of each of the six primers, 1:1 TEMPase HotStart Master Mix (Ampliqon-Denmark), and 3 μL of extracted DNA. The cycling conditions were as follows: preheating at 95°C for 15 min, followed by 35 cycles of denaturation at 94°C for 30 s, annealing at 50°C for 30 s, extension at 72°C for 60 s, and a final extension at 72°C for 5 min. Amplified DNA of the sibling species of *An. gambiae* complex and *An. funestus* group was separated based on their fragment size using 1.5% agarose gel electrophoresis and visualized under ultraviolet light. The expected fragment sizes for the prevalent sibling species of the *An. gambiae* complex are 153 bp for *An. quadriannulatus*, 466 bp for *An. merus*, 390 bp for *An. gambiae s.s*, and 315 bp for *An. arabiensis* [37]. For sibling species of the *An. funestus* group, the expected fragment sizes are 587 bp for *An. vaneedeni*, 505 bp for *An. funestus* s.s, 411 bp for *An. rivulorum*, 252 bp for *An. parensis*, and 146 bp for *An. leesoni* [38].

#### Detection of *Plasmodium falciparum* infection.

DNA was extracted from the head and thorax of *An. gambiae* complex and *An. funestus* group and analyzed using quantitative real-time PCR (qPCR) to detect *P. falciparum* infection. The qPCR assay targeted the 18S ribosomal subunit and was performed as previously described [39]. This method was validated with that of Bass and colleagues [40] by running a parallel test on 59 *Anopheles* specimens (20 *An. gambiae* s.s, 20 *An. funestus* s.s and 19 *An. arabiensis*) with known infectivity. The findings from the two methods agreed perfectly (κ = 0.946) and the former was used for subsequent testing. Briefly, qPCR was conducted in a final volume of 13 μL, consisting of 1.25 μL of nuclease-free water (BioConcept, Paradiesrain, Switzerland), 0.8 μM of each primer (Integrated DNA Technologies Inc., Coralville, IA, USA), 0.4 μM of probes (Integrated DNA Technologies Inc., Coralville, IA, USA), 2x Universal Probe Master mix (BioRad Laboratories, USA) and 3.0 μL of genomic DNA. Samples were run on a CFX Opus 96 Real-Time PCR system (BioRad Laboratories, Hercules, CA, USA), with the following thermal profile: 2 min at 50°C, 10 min at 95°C, and 45 cycles at 95°C for 9 seconds, and 55°C for 1 min.

### Data analysis

Data were collected using the MSMT tools deployed on smartphones, implemented using the Open Data Kit software (ODK), and subsequently transferred to a remote server at the National Institute for Medical Research. The data were downloaded to a Microsoft Excel spreadsheet and cleaned before the analysis. The human biting rate of the mosquitoes was calculated as the number of female mosquitoes per trap- night for each collection method. Mosquito infection with *P. falciparum* by qPCR, a proxy for sporozoite rate, was estimated as the proportion of mosquitoes positive for *P. falciparum* over the total number tested. The annual entomological inoculation rate (EIR) was calculated from mosquitoes collected with CDC light traps using the formula: 1.605 × (number of *P. falciparum* positive qPCR/ number of mosquitoes tested) × (number of mosquitoes collected/number of trap-nights) × 365 [41]. The multiplication factor of 1.605 is a conversion value used to compare CDC light trap catches with human biting catches, as previously described [42]. The estimated EIR between districts was compared using a joint Wald test statistic, and a p-value ≤ 0.05 was considered statistically significant.

### Ethical considerations

This study was approved by the Medical Research Coordinating Committee of the National Institute for Medical Research, Tanzania (Reference number NIMR/HQ/R.8a/Vol. 1X/3579 dated 16^th^ December 2020) as part of the MSMT Project. Before mosquito collection, meetings were held with the district and the respective village leaders to inform them about the study and obtain their cooperation. Written informed consent was obtained from the heads of households before mosquito collection commenced in their respective houses.

## Results

### Mosquito abundance and species composition

**Fig 3:**
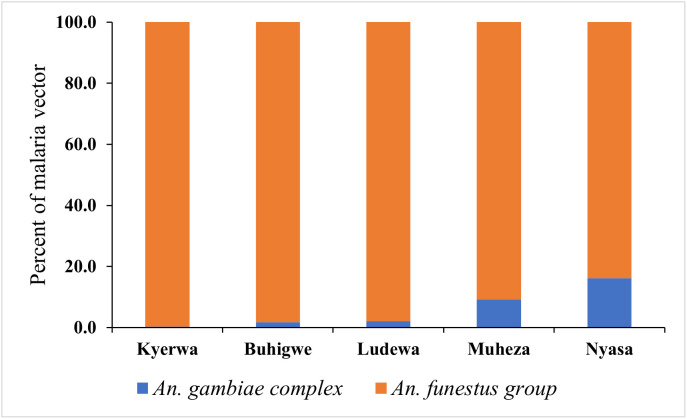
Composition of malaria vectors collected by different trap types in five surveyed districts.

A total of 19,898 adult mosquitoes were collected from the five districts: Buhigwe (Kigoma region), Kyerwa (Kagera region), Ludewa (Njombe region), Muheza (Tanga region) and Nyasa (Ruvuma region). Among the collected mosquitoes, *An. gambiae* complex, *An. funestus* group, other *Anopheles* species and culicine species accounted for 2.2% (n = 431), 44.3% (n = 8812), 1.1% (n = 226) and 52.4% (n = 10,429), respectively. Because other *Anopheles* species were collected in low numbers, they were not identified further and were grouped with culicine species in the subsequent presentation. Overall, more mosquitoes were collected in Kyerwa district (80.5%, n = 16,012). Of all the malaria vectors collected, 91.9% (n = 396) of *An. gambiae* complex were collected in the Nyasa district, whereas 71.1% (n = 6262) of *An. funestus* group were collected from the Kyerwa districts (Table 1, Fig 3). Analysis of mosquitoes in the study villages revealed that the majority of mosquitoes were collected from Kitoma, Rubuga, and Ruko in the Kyerwa district (Table 2). More mosquitoes were collected indoors using CDC light traps than outdoors using the Furvela tent traps (Table 3). The human biting rate was more intense in Kyerwa, where, on average, 69 and 72 mosquitoes were collected per trap per night outdoors and indoors, respectively (Table 3). All mosquitoes collected by the Furvela tent trap were found to be unfed. Likewise, the majority (92.1%) of *An. gambiae* complex, and 86.1% of *An. funestus* group collected using the CDC light traps were unfed (Table 4). A high proportion (93.4%) of the unfed *An. funestus* group were collected from Nyasa district. Compared with other districts, approximately 15 percent (14.5%) of *An. funestus* group collected from Muheza district were blood-fed (Table 4).

**Table 1:** Total number and percentage (%) of female mosquito species collected indoors and outdoors from five surveyed districts.

Region	District	Total collected	*An. gambiae* complex (%)	*An. funestus* group (%)	Other *Anopheles* (%)	^ǂ^Culicine species (%)
Kigoma	Buhigwe	554	7 (1.3)	384 (69.3)	3 (0.5)	160 (28.9)
Kagera	Kyerwa	16012	20 (0.1)	6262 (39.1)	148 (0.9)	9582 (59.9)
Njombe	Ludewa	75	1 (1.3)	46 (61.3)	2 (2.7)	26 (34.7)
Tanga	Muheza	175	7 (4.0)	69 (39.4)	0 (0.0)	99 (56.6)
Ruvuma	Nyasa	3082	396 (12.8)	2051 (66.5)	73 (2.4)	562 (18.2)
**Total**		**19898**	**431 (2.2)**	**8812 (44.3)**	**226 (1.1)**	**10429 (52.4)**

ǂ Culicine species comprised *Culex*, *Aedes*, and *Mansonia* species

**Table 2:** Total number and percentage (%) of female mosquitoes collected indoors and outdoors by village.

District	Village	Total	*An. gambiae* complex (%)	*An. funestus* group (%)	Other mosquitoes (%)
Buhigwe	Nyankoronko	265	3 (1.1)	125 (47.2)	137 (51.7)
	Kigege	289	4 (1.4)	259 (89.6)	26 (9.0)
Ludewa	Kipangala	75	1 (1.3)	46 (61.3)	28 (37.3)
Nyasa	Lipingo	315	38 (12.1)	141 (44.8)	136 (43.2)
	Ngindo	735	143 (19.5)	333 (45.3)	259 (35.2)
	Lundo	2017	213 (10.6)	1577 (78.2)	227 (11.3)
	Chiulu	15	2 (13.3)	0 (0.0)	13 (86.7)
Muheza	Mamboleo	66	3 (4.5)	26 (39.4)	37 (56.1)
	Magoda	41	3 (7.3)	0 (0.0)	38 (92.7)
	Mpapayu	68	1 (1.5)	43 (63.2)	24 (35.3)
Kyerwa	Kitwechenkura	1527	6 (0.4)	430 (28.2)	1091 (71.4)
	Nyakabwera	1230	6 (0.5)	8 (0.7)	1216 (98.9)
	Kitoma	4901	3 (0.1)	2668 (54.4)	2230 (45.5)
	Rubuga	4634	3 (0.1)	1878 (40.5)	2753 (59.4)
	Ruko	3720	2 (0.1)	1278 (34.4)	2440 (65.6)

**Table 3:** Total abundance and human biting rate (in parentheses) of mosquitoes collected by different trap types in five surveyed districts.

District	Furvela tent traps				CDC light traps			
	*An. gambiae* complex	*An. funestus* group	Other mosquitoes	Total Mosquitoes	*An. gambiae* complex	*An. funestus* group	Other mosquitoes	Total Mosquitoes
Buhigwe	0 (0.00)	37 (1.23)	85 (2.83)	122 (4.07)	7 (0.12)	347 (5.78)	78 (1.03)	432 (7.20)
Kyerwa	9 (0.12)	970 (12.93)	4191 (55.88)	5170 (68.93)	11 (0.07)	5292 (35.28)	5539 (36.93)	10842 (72.28)
Ludewa	0 (0.00)	1 (0.07)	5 (0.33)	6 (0.40)	1 (0.03)	45 (1.50)	23 (0.77)	69 (2.30)
Muheza	0 (0.00)	0 (0.00)	13 (0.29)	13 (0.29)	7 (0.08)	69 (0.77)	86 (0.96)	162 (1.80)
Nyasa	169 (2.82)	206 (3.43)	161 (2.68)	536 (8.93)	227 (1.89)	1845 (15.38)	474 (3.95)	2546 (21.22)
**Total**	**178 (0.79)**	**1214 (5.40)**	**4455 (19.80)**	**5847 (25.99)**	**253 (0.56)**	**7598 (16.88)**	**6200 (13.78)**	**14051 (31.22)**

**Table 4:** Physiological status of malaria vectors collected with CDC light traps in five surveyed districts.

District	*An. gambiae complex*				*An. funestus group*			
	Total	Unfed, n (%)	Fed, n (%)	Gravid, n (%)	Total	Unfed, n (%)	Fed, n (%)	Gravid, n (%)
Buhigwe	7	7 (100)	0 (0)	0 (0)	347	314 (90.5)	21 (6.1)	12 (3.5)
Kyerwa	11	11 (100)	0 (0)	0 (0)	5292	4404 (83.2)	597 (11.3)	291 (5.5)
Ludewa	1	1 (100)	0 (0)	0 (0)	45	39 (86.7)	5 (11.1)	1 (2.2)
Muheza	7	7 (100)	0 (0)	0 (0)	69	59 (85.5)	10 (14.5)	0 (0.0)
Nyasa	227	207 (91.2)	18 (7.9)	2 (0.9)	1845	1724 (93.4)	97 (5.3)	24 (1.3)
**Total**	**253**	**233 (92.1)**	**18 (7.1)**	**2 (0.8)**	**7598**	**6540 (86.1)**	**730 (9.6)**	**328 (4.3)**

#### Identification of sibling species of *An. gambiae* complex and *An. funestus* group.

A total of 1799 (approximately 20% of the collected malaria vectors) were selected and processed for sibling species identification by PCR. The 20% sub-sampling threshold has been found generally reliable in large samples of insects [43]. Out of 1799 sampled vectors, nearly all collected *An. gambiae complex* (n = 408, 22.7%) were included, while 1391 (77.3%) *An. funestus* group specimens were randomly chosen from 8812 collected samples. A total of 1764 (98.1%) vectors were successfully genotyped into the sibling species of *An. gambiae* complex (n = 404), and *An. funestus* group (n = 1360). Among the 404 identified, *An. gambiae* complex specimens, three-quarters (75.0%, n = 303) were *An. arabiensis* and the remaining (25.0%, n = 101) were *An. gambiae* s.s sibling species (Table 5). On the other hand, of the 1360 identified specimens of *An. funestus* group, the majority were *An. funestus* s.s (96.3%, n = 1310), followed by *An. parensis* (1.8%, n = 25), *An. rivulorum* (1.1%, n = 15) and *An. leesoni* (0.7%, n = 10) (Table 6).

**Table 5:** Composition of *An. gambiae* complex collected from the five surveyed districts.

District	No. Tested	No. PCR identified (%)	No. (%) sibling species identified	
			*An. arabiensis*	*An. gambiae s.s*
Buhigwe	7	6 (85.7)	2 (33.3)	4 (66.7)
Kyerwa	19	18 (94.7)	15 (83.3)	3 (16.7)
Ludewa	1	1 (100.0)	1 (100.0)	0 (0.0)
Muheza	7	7 (100.0)	3 (42.9)	4 (57.1)
Nyasa	374	372 (99.5)	282 (75.8)	90 (24.2)
Total	**408**	**404 (99.0)**	**303 (75.0)**	**101 (25.0)**

**Table 6:** Composition of *An. funestus* group collected from the five surveyed districts.

District	No. tested	No. PCR identified (%)	No. sibling species identified (%)			
			*An. funestus s.s*	*An. parensis*	*An. leesoni*	*An. rivulorum*
Buhigwe	123	114 (92.7)	112 (98.3)	0 (0.0)	2 (1.8)	0 (0.0)
Kyerwa	697	684 (98.1)	661 (96.6)	23 (3.4)	0 (0.0)	0 (0.0)
Ludewa	37	35 (94.6)	31 (88.6)	0 (0.0)	4 (11.4)	0 (0.0)
Muheza	69	65 (94.2)	65 (100.0)	0 (0.0)	0 (0.0)	0 (0.0)
Nyasa	465	462 (99.4)	441 (95.5)	2 (0.4)	4 (0.9)	15 (3.3)
Total	**1391**	**1360 (97.8)**	**1310 (96.3)**	**25 (1.8)**	**10 (0.7)**	**15 (1.1)**

#### Malaria vector infection with *P. falciparum.*

All 1799 malaria vector specimens examined for sibling species identity were screened for infection with *P. falciparum*. Of the vectors tested for *P. falciparum* infection, 1126 (62.6%) were collected using CDC light trap and the rest (n = 673, 37.4%) were collected using a Furvela tent trap. Nine malaria vectors were found to be infected with *P. falciparum* parasites, all of which belonged to *An. funestus* s.s (Table 7). None of the malaria vectors collected using the Furvela tent trap were found to be infected. *P. falciparum* infected mosquitoes were collected in Kyerwa (n = 5), Nyasa (n = 2) and Muheza (n = 2). The overall *P. falciparum* sporozoite rate was 0.5% for all vectors tested whereas for *An. funestus* group, the sporozoite infection was 0.66%. Based on CDC light trap collection, the respective annual entomological inoculation rate (EIR) for Kyerwa, Nyasa, and Muheza was 242.6, 66.5, and 13.0 infectious bites per person per year. The estimated annual EIR differed significantly across the surveyed districts (p < 0.001). The distribution of *P. falciparum*-infected mosquitoes, sporozoite rates, and annual EIR are presented in Table 7.

**Table 7:** Distribution of *P. falciparum*-infected mosquitoes, sporozoite rate, and annual EIR for CDC light trap catch.

District	Malaria Vector	No. Collected	Trap-nights	Biting rate	No. tested for *P. falciparum*	No. infected	Sporozoite rate	Annual EIR (95% CI)
Buhigwe	*An. gambiae* complex	7	60	0.12	7	0	0.00	0.00 (0.00 - 0.00)
	*An. funestus* group	347	60	5.78	88	0	0.00	0.00 (0.00 - 0.00)
Kyerwa	*An. gambiae* complex	11	150	0.07	13	0	0.00	0.00 (0.00 - 0.00)
	*An. funestus* group	5292	150	35.28	426	5	0.01	242.58 (31.2-459.9)
Ludewa	*An. gambiae* complex	1	30	0.03	1	0	0.00	0.00 (0.00 - 0.00)
	*An. funestus* group	45	30	1.50	37	0	0.00	0.00 (0.00 - 0.00)
Muheza	*An. gambiae* complex	7	90	0.08	7	0	0.00	0.00 (0.00 - 0.00)
	*An. funestus* group	69	90	0.77	69	2	0.03	13.02 (0.00–31.1)
Nyasa	*An. gambiae* complex	227	120	1.89	207	0	0.00	0.00 (0.00 - 0.00)
	*An. funestus* group	1845	120	15.38	271	2	0.01	66.47 (0.00 −158.6)
Total	*An. gambiae* complex	253	450	0.56	235	0	0.00	0.00 (0.00 - 0.00)
	*An. funestus* group	7598	450	16.88	891	9	0.01	99.91 (34.9–164.9)

CI = confidence interval.

## Discussion

The persistent high malaria burden in some SSA countries is caused by the presence of efficient vectors, and the disease in this region is mainly caused by *P. falciparum*, which is a more deadly malaria parasite than other *Plasmodium* species [44]. As efforts to control the disease intensify, vectors are changing their behavior (biting and resting) and have also become resistant to most classes of insecticides used for their control [13,17,18]. On the other hand, the threat of antimalarial drug resistance is increasing at unprecedented levels, with recent reports of partial artemisinin resistance, which has been confirmed in East Africa (Rwanda, Uganda, and Tanzania) and the Horn of Africa (in Eritrea), and parasites carrying resistance mutations in Ethiopia, Sudan, and Kenya [45,46]. These developments in malaria vectors and the parasites they transmit are compounded by the effects of climate change, which creates favorable conditions for vector and parasite propagation [47]. Furthermore, traditional malaria control challenges are amplified by the impending threat of invasion by *An. stephensi*, which has been reported in some SSA countries [48]. These control challenges call for concerted efforts in malaria control to sustain the gains achieved so far and accelerate towards the goal of malaria elimination.

Malaria entomological surveillance provides the information required to facilitate the implementation of appropriate vector control operations. Mosquito surveillance during the dry season provides information on an important segment of the population that exhibit unique survival trait that allows them to survive harsh dry conditions. This population underlies the persistence of the mosquito population and is the base stock for the rebound of mosquitoes during the rainy season. CDC light traps have been considered an effective tool for the collection of host-seeking mosquitoes and compare favorably with the human landing catch, a standard method for sampling host-seeking mosquitoes [42]. Furvela tent traps have been recommended for the collection of outdoor biting malaria vectors [34]. Using these two trap types, this study documented the relative abundances of *An. gambiae* complex and *An. funestus* group in five districts from five regions, some of which reported persistent malaria transmission in mainland Tanzania [25]. The findings revealed that *An. funestus* group was the predominant malaria vector collected from all surveyed sites. This finding corroborates previous evidence from several reports indicating the re-emergence of *An. funestus* as an important malaria vector in Tanzania [9,10]. A recent review of relevant articles from Eastern and Southern Africa revealed that the role of *An. gambiae* has declined and *An. funestus* has become the predominant malaria vector in East and Southern Africa [49].

Molecular identification of sibling species of *An. gambiae* complex revealed the presence of *An. gambiae* s.s and *An. arabiensis* in the study areas. *An. arabiensis* was the predominant sibling species, accounting for three-quarters (75.0%) of members of *An. gambiae* complex. A relatively high population of *An. arabiensis* recorded is supported by a recent study documenting the predominance of this sibling species in mainland Tanzania [26]. On the other hand, the analysis of *An. funestus* group revealed that *An. funestus* s.s was the predominant (96.3%) sibling species, whereas the *An. parensis*, *An. rivulorum*, and *An. leesoni* were also found in the study areas. These four sibling species are commonly reported in many areas of Tanzania [9,26]. *An. funestus* s.s is the most predominant member in number and distribution among the sibling species of *An. funestus* groups in Tanzania and beyond [7]. These findings and published results from other studies have revealed an increase in the potential role of the *An. funestus* in malaria transmission in many settings in East and Southern Africa [26,49].

Screening of malaria vectors for infection with *P. falciparum* revealed nine (9) infected malaria vectors, with an overall infection rate of 0.5% in 1799 examined specimens. *P. falciparum* infection was detected only in *An. funestus* s.s collected using CDC light traps, with an infection rate of 0.66%. Based on the estimated annual EIR, malaria transmission intensity was relatively higher in the Kyerwa district of Kagera region, followed by Nyasa (Ruvuma) and Muheza (Tanga), with 242.6, 66.5 and 13.0 infectious bites per person per year, respectively. According to the malaria epidemiological stratification in mainland Tanzania, four of the five surveyed districts (Kyerwa, Buhigwe, Nyasa, and Muheza) were categorized as high malaria transmission strata [50]. The relatively high annual EIR recorded in Kyerwa agrees with the high malaria prevalence (44.4%) reported in a survey conducted just before mosquito collections in the same surveyed villages [31]. Furthermore, a recent entomological study conducted in a district bordering Kyerwa (Missenyi) between 2018 and 2022 recorded high malaria transmission intensity, with an EIR of 108.3 infectious bites per person per year [51]. The recorded EIR in the surveyed districts is far beyond the < 1 infectious bite per person per year recommended to achieve the national target of malaria elimination by 2030 [50].

The analysis of the physiological status of the collected mosquitoes documented up to 10% of blood-fed mosquitoes in the CDC light trap catch from the surveyed districts, suggesting that individuals in the study communities were not optimally protected by existing LLINs interventions. A countrywide malaria indicator survey conducted in 2022 indicated a slight decline in household ownership of LLINs from 78% in 2017 to 74% in 2022 [25,52]. In the same surveillance, LLINs use by children below 5 years was reported to be 64%, which agreed with the LLINs use of 63.9% recently reported in Kyerwa district [31]. Of particular concern, IRS intervention, which was providing additional protection in the lake zone and other regions of Tanzania, has been withdrawn since 2022 [53]. IRS is an important strategy for insecticide resistance management by deploying insecticide classes different from those impregnated in LLINs. The WHO recommends rotation of IRS insecticides with different modes of action at predetermined time intervals to manage insecticide resistance [12]. The findings of relatively high mosquito feeding success in the study areas call for the deployment of complementary mosquito control interventions to supplement the limitations of existing LLINs.

In this study, a substantial number of malaria vectors were collected both indoors and outdoors. The human biting by the malaria vector was intense, reaching 35 and 13 *An. funestus* group per trap per night indoors and outdoors, respectively, in one of the surveyed districts. *An. funestus* is known to be highly anthropophilic, endophilic, and resistant to commonly used insecticides [7]. Other studies have demonstrated that the emergence and widespread insecticide resistance result in suboptimal community protection by LLINs and IRS in different endemic areas of Tanzania [54]. Over the past two decades, the prevalence and intensity of insecticide resistance have increased significantly in Tanzania, affecting most classes of insecticides of public health importance, and have been recorded in all malaria vector species [15,55]. The current findings of relatively high biting rates observed both indoors and outdoors, the reported behavior adaptation by malaria vectors [17,23] and insecticide resistance [15,54,55] may be the underlying reasons for the increased risk of malaria transmission at the study sites.

To accelerate malaria elimination efforts, the current control interventions need to be complemented with novel methods in an integrated manner to further reduce malaria burden to meet national malaria control and elimination targets. Larval source management is an important complementary strategy that has proven to be useful for inclusion in integrated vector management operations with the potential to control both indoor and outdoor biting mosquitoes, as well as mosquitoes with resistance genotypes [56,57]. To mitigate insecticide resistance, there is a need to reconsider the implementation of IRS and deployment of next-generation LLINs as priority interventions in districts with high malaria burden.

The current study was conducted over a relatively short period and focused on MSMT sites, selected as previously described [29]. The absence of *P. falciparum*-infected mosquitoes in Ludewa (moderate transmission strata) and Buhigwe (high transmission strata) remains inconclusive and highlights the need for longitudinal entomological surveillance covering both wet and dry seasons. A longitudinal study could produce more robust and comprehensive entomological data to inform malaria vector control strategies. Despite these limitations, our findings support previous reports on the composition and infectivity of malaria vectors in these regions [26,49,51] and hold local or even regional relevance for malaria control.

## Conclusions

This study reported a relatively high density of malaria vectors in the study areas, despite being conducted during the dry season only. The findings suggest that, in most of the surveyed districts, *An. funestus* is the major malaria vector, and interventions targeting this vector may have a great impact on reducing malaria transmission. Moreover, the collection of a substantial number of blood-fed mosquitoes in light trap catches indicated that the community members were not fully protected by the core malaria vector control interventions, which calls for the adoption of integrated vector management strategies in mosquito control.
